# Toxicological Effects and Mechanisms of 2,2′,4,4′-Tetrabromodiphenyl Ether (BDE-47) on Marine Organisms

**DOI:** 10.3390/toxics12100747

**Published:** 2024-10-15

**Authors:** Boyang Li, Yun Shao, Chen Liu, Jie Wang, Yanzhong Zhu, Xiaoqian Li

**Affiliations:** 1 Southern Marine Science and Engineering Guangdong Laboratory (Zhuhai), Zhuhai 519000, China; lby1969648319@163.com; 2 State Key Laboratory of Environmental Criteria and Risk Assessment, Chinese Research Academy of Environmental Sciences, Beijing 100012, China; sy.sherin@outlook.com (Y.S.); liuchen--@outlook.com (C.L.); wangjiew2021@163.com (J.W.); 3 National Joint Research Center for Yangtze River Conservation, Chinese Research Academy of Environmental Sciences, Beijing 100012, China

**Keywords:** BDE-47, marine organisms, toxic effects, toxicity mechanisms

## Abstract

2,2′,4,4′-tetrabromodiphenyl ether (BDE-47) is a widely used brominated flame retardant belonging to persistent organic pollutants (POPs). After being released into the marine environment, BDE-47 can cause a range of toxic effects on marine organisms through bioaccumulation, biomagnification, and intergenerational transmission. These effects include lethality, impaired motility, photosynthetic toxicity, immune damage, liver toxicity, developmental impairments, and reproductive toxicity. This article reviews the latest research progress on the toxic effects and molecular mechanisms of BDE-47 mentioned above. The primary mechanisms underlying its toxicity include oxidative stress, DNA damage, cellular apoptosis, impaired metabolism, and activation of the MAPK signaling cascade.

## 1. Introduction

2,2′,4,4′-tetrabromodiphenyl ether (BDE-47) is a brominated flame retardant widely used as an additive in various consumer goods, including foaming plastics and electronic appliances [1]. According to the Stockholm Convention, polybrominated diphenyl ethers (PBDEs) are classified persistent organic pollutants (POPs), and the ocean serves as a significant sink for them [2]. Previous studies have found significant differences in the patterns and concentrations of PBDEs in seawater compared to freshwater organisms [3]. Specifically, marine organisms often accumulate higher levels of PBDEs due to longer food chains and higher pollution, leading to more pronounced biomagnification. In contrast, freshwater organisms generally have lower PBDE levels because of shorter food chains and lower environmental concentrations, though they can still suffer from toxic effects such as growth inhibition and reproductive issues. These differences are due to the distinct environmental conditions and biological processes in marine and freshwater ecosystems.

Recently, increased concentrations of PBDEs have been detected in the environment [4,5] and the organisms [6,7]. The marine ecosystem has been heavily contaminated, with widespread detection observed in wild marine organisms [8,9], especially in the fat and liver tissues [10,11]. Research indicates a substantial accumulation of PBDEs in the rare green sea turtle (*Chelonia mydas*), seemingly causing toxicity similar to dioxins [12]. Additionally, these substances were commonly detected in fish [13], primarily accumulating in the liver and muscles [14]. Furthermore, they were widely identified in bivalves [15,16,17], with the total concentration in purple clams ranging from 0.2 to 6.9 ng/g (dry weight) [18]. In the last decade, PBDEs have been found in peoples’ diets [19], leading to a series of implications for human health [20]. The most significant and active component among the detected PBDEs in biological samples is BDE-47 [21,22,23,24], typically accounting for 70% of the total polybrominated diphenyl ether content [25].

In fact, in marine environments, BDE-47 is considered one of the most bioavailable congeners among polybrominated diphenyl ethers [2,26]. BDE-47 exhibits a high Bioconcentration Factor (BCF) and has time-dependent bioaccumulation, with a long half-life in water [27]. Apart from seawater, BDE-47 in marine sediments stands out as a key source of this compound within marine organisms [28,29]. Through processes like dry and wet deposition, BDE-47 can extend its reach to remote areas as well, subsequently entering the marine food web [30].

BDE-47, which is classified as a low-brominated polybrominated diphenyl ether, exhibits significantly higher toxicity compared to other congeners. A significant correlation has been observed between the pollution concentration of BDE-47 in the blubber of gray seal pups and their first-year survival rate [31]. A research finding suggested that its toxicity to marine rotifers (*Brachionus plicatilis*) is 13 times higher than that of the highly brominated congener BDE-209 [32]. BDE-47 accumulates within organisms, undergoing biomagnification within the food web. The bioconcentration factors (BCFs) of tetrabrominated to hexabrominated diphenyl ethers in the marine food web can reach 17–76, indicating their strong bioaccumulative potential [33], and BDE-47 remains the predominant congener [34]. Additionally, it undergoes debromination reactions within organisms, resulting in increased toxicity, which is a phenomenon known as biomagnification [35].

In recent years, the application of advanced bioanalytical techniques has led to the discovery of numerous mechanisms of toxicity caused by BDE-47 in seawater, which; however, have not been extensively elaborated upon in the current review literature. Currently, there is a limited preliminary assessment of the baseline for BDE-47 in the marine environment [36]. Given the diversity and strong bioaccumulative nature of the toxic effects of BDE-47, a comprehensive consideration of various toxicity aspects is necessary to establish a more robust baseline for BDE-47 in the marine environment. Therefore, considering insights from recent research, this review summarizes the toxic effects and the associated mechanisms of BDE-47 on marine organisms.

## 2. Toxic Effects of BDE-47 to Marine Organisms

### 2.1. Lethality

Only a few studies have investigated the median lethal concentrations (LC_50_) of BDE-47 in marine organisms, and the values decrease with increasing exposure time (Table 1). Sensitivity to BDE-47 ranges considerably among different species. In the studies currently compiled, comparing the 96 h LC_50_, one marine flea, the *Eurytemora pacifica*, exhibits the lowest tolerance, with a value of only 57 µg/L, while another harpacticoid copepod species, *Tigriopus japonicas*, belonging to the same order, has the highest LC_50_ at 851 µg/L [37].

### 2.2. BDE-47-Induced Morphological Damage

BDE-47 exhibits multifaceted toxicity to marine organisms in different nutrient levels, including phylum of *Bacillariophyta*, *Chlorophyta*, *Dinophyta*, *Rotifera* and *Mollusca*. The effects and the mechanisms involved are collated in Table 2. BDE-47 at different concentrations may directly damage the morphological structure of biological cells. Zhang et al. [40] found that *Skeletonema costatum* exhibited slight deformation at 100 µg/L BDE-47 treatment. As the concentration increased, the pillar processes on the upper and lower epithelia began to separate. At 600 µg/L, the girdle bands markedly contracted and detached from the vesicle membrane, and the pillar processes on both the upper and lower epithelia completely separated.

The extent of morphological damages varies among different algal species exposed to the same concentration of BDE-47. A research study conducted by Zhao et al. [39] compared the morphological damage under exposure for 120 h in *Alexandrium minutum* and *Dunaliella salina*. They found that the membrane system and cell organelles of *A. minutum* were more damaged compared to *D. salina*. Jian et al. [41] conducted acute toxicity experiments by exposing 2 h old newly hatched *Brachionus plicatilis* to BDE-47. The shape of normal rotifers cultivated in sterilized seawater was round and intact, and the internal organs were clear. However, individuals exposed to BDE-47 at a concentration of 2 mg/L for 96 h appeared dark, and the internal structure was hard to distinguish. In addition, the caudal leg lagged or stuck to the bottom of the well plate. The form of the organisms changed to pear-shaped, and the trochal disk and carapace appeared to shrink with exposure. In the study by Geng et al. [42], significant hemolysis infiltration accompanied by mild fibrosis was observed in the gill and digestive gland sections of blue mussels (*Mytilus edulis*) exposed to 10 µg/L for 16 days. Additionally, there was an increase in the thickness of atrophied tubules, representing an inflammatory response, along with an enlargement of the tubular lumen.

BDE-47 can cause significant damage to the ultrastructure of biological cells even under low concentrations and short-term exposure. In algae, this primarily manifests as damage to chloroplasts, mitochondrial membrane, and the appearance of cytoplasmic vacuolization [39,43,44]. In other types of cells, dissolution of cells and an increase in blood granules and vesicles are predominant effects [45,46]. Apart from that, sublethal concentrations of BDE-47 also lead to damage in the ultrastructure of ovaries [47], potentially further impacting the reproductive capabilities of organisms. Observations at just 8 µg/L for 24 h revealed wrinkling of follicles and deformation of nuclear membranes in *Brachionus plicatilis* [48].

**Table 2:** Toxic effects and related mechanisms of BDE-47 on marine organisms.

Phylum	Species	Concentrations	Life Stage	Duration of Exposure	Exposure Pathways	Primary Targets	Toxic Effects	Mechanisms	Ref.
*Chlorophyta*	*Chlorella* sp.	60, 120 µg/L	--	96 h	immersion	--	impaired morphology, photosynthesis toxicity, inhibited population growth	oxidative stress, PCD, excessive produced Ca^2+^	[43]
		0.1, 1.0, 2.5 µg/L	--	96 h	immersion	--	photosynthesis toxicity	--	[49]
		0.1, 1.0, 2.5 µg/L	--	96 h	immersion	--	photosynthesis toxicity	--	[49]
	*Chlorella autotrophica*	0, 0.1, 1, 5, 10, 50 µg/L	--	96 h	immersion	--	inhibited population growth	--	[50]
		0.1, 1.0, 2.5 µg/L	--	96 h	immersion	--	--	oxidative stress	[51]
	*Chlorella pyrenoidosa*	0.5, 1, 2, 4, 8 µg/L	--	96 h	immersion	--	inhibited population growth	--	[52]
	*Platymonas subcordiformis*	0.5, 1, 2, 4, 8, 16, 32 µg/L	--	2 h	immersion	--	impaired motility	--	[21]
		1, 10, 50, 100, 300 µg/L	--		immersion	--	inhibited population growth	--	[53]
		1, 10, 50, 100, 300 µg/L	--		immersion	--	inhibited population growth	--	[53]
	*Dunaliella salina*	1, 10, 50, 100, 300 µg/L	--		immersion	--	inhibited population growth	--	[53]
		1, 10, 50, 100, 300 µg/L	--		immersion	--	inhibited population growth	--	[53]
		0.1, 0.5, 1 mg/L	--	120 h	immersion	--	impaired morphology, photosynthesis toxicity, developmental toxicity	oxidative stress	[39,54]
		0.1, 0.5, 1 mg/L	--	120 h	immersion	--	impaired morphology, photosynthesis toxicity, developmental toxicity	oxidative stress	[39,54]
*Bacillariophyta*	*Thalassiosira pseudonana*	25 µg/L	--	24 h	immersion	--	--	whole transcriptome resequencing	[55]
		25 µg/L	--	120 h	immersion	--	--	oxidative stress, PCD	[56]
		5, 15, 25 µg/L	--	96 h	immersion	--	inhibited population growth	DNA damage	[57]
	*Skeletonema costatum*	50, 100, 200, 400, 600 µg/L	--	96 h	immersion	--	impaired morphology, photosynthesis toxicity, inhibited population growth	oxidative stress	[40]
		0, 0.1, 1, 5, 10, 50 µg/L	--	96 h	immersion	--	inhibited population growth	--	[50]
		0.1, 1.0, 2.5 µg/L	--	96 h	immersion	--	--	oxidative stress	[51]
		0.16, 0.31, 0.63, 1.25, 2.50 mg/L	--	96 h	immersion	--	inhibited population growth	--	[58]
		1.0, 3.2, 10, 32, 100 µg/L	--	96 h	immersion	--	inhibited population growth	--	[59]
	*Phaeodactylum tricornutum*	0.8, 2, 4, 6, 8 mg/L	--	96 h	immersion	--	impaired morphology, photosynthesis toxicity	oxidative stress	[44]
	*Chaetoceros muelleri*	0, 0.1, 1, 5, 10, 50 µg/L	--	96 h	immersion	--	inhibited population growth	--	[50]
		0.1, 1.0, 2.5 µg/L	--	96 h	immersion	--	--	oxidative stress	[51]
*Dinoflagellata*	*Alexandrium minutum*	0.1, 0.5, 1 mg/L	--	120 h	immersion	--	impaired morphology, photosynthesis toxicity, developmental toxicity	oxidative stress	[39,54]
	*Heterosigma akashiwo*	0.1, 1.0, 2.5 µg/L	--	96 h	immersion	--	photosynthesis toxicity	--	[49]
		0, 0.1, 1, 5, 10, 50 µg/L	--	96 h	immersion	--	inhibited population growth	--	[50]
		0.1, 1.0, 2.5 µg/L	--	96 h	immersion	--	--	oxidative stress	[51]
*Rotifera*	*Brachionus plicatilis*	0.8, 2, 6, 10, 14, 18, 22 mg/L	0–2 h	24–96 h	immersion	the whole body	inhibited population growth, impaired motility, reproductive toxicity	--	[32]
		2.0, 6.0, 10, 14, 18, 22 mg/L	0–2 h	24 h	immersion		impaired motility	--	[38]
		2, 6, 10, 14, 18, 22 mg/L	2 h	96 h	immersion	the whole body	impaired morphology, inhibited population growth, reproductive toxicity	oxidative stress, activation of the detoxification system	[41]
		0.08, 0,8 and 8 mg/L	female adults	24 h	immersion	stomach and ovary	impaired morphology	ROS, apoptosis, mitophagy	[47]
		0.008, 0.08, 0.8 mg/L	0–2 h	0–200 h	immersion	ovary	impaired morphology, reproductive toxicity	oxidative stress	[60]
		0.02, 0.1, 0.5 mg/L	amictic females	24 h	immersion	the whole body	--	oxidative stress, DNA damage, apoptosis, Metabolic disorders (Metabolomics)	[61]
		31.25, 125, 500 µg/L	2 h	24 h	immersion	stomach	impaired morphology, impaired feeding	impaired feeding and metabolism	[47,62]
		0.1, 0.2, 0.5, 1.0, 2.0, 3.9, 7.8, 15.6, 31.3, 62.5, 125.0 mg/L	2 h	72 h	immersion	--	inhibited population growth	--	[63]
		0.05, 0.1, 0.2 mg/L	0–2 h	17 d	immersion	ovary	reproductive toxicity, developmental toxicity	down-regulated vasa mRNA	[64]
		0.05, 0.1, 0.2 mg/L	adult	24 h	immersion	ovary	reproductive toxicity	oxidative stress, excessive produced Ca^2+^	[65]
		0.0008, 0.008, 0.08, 0.8, 2, 4, 8 mg/L	0–2 h	full life history	immersion	--	inhibited population growth, developmental toxicity	--	[66]
*Arthropoda*	*Tigriopus japonicus*	0.7125, 1.425, 2.85, 5.7, 11.4 µg/L; 10.6375, 21.275, 42.55, 85.11, 170.2 µg/L	adult	96 h	immersion	the whole body	24 h impaired feeding	oxidative stress	[37]
		0.05, 60, 120 µg/L	less than 12 hph nauplii	20 d	immersion	the whole body	reproductive toxicity, developmental toxicity	oxidative stress, activation of the detoxification system, apoptosis, DNA damage	[67]
	*Eurytemora pacifica*	0.7125, 1.425, 2.85, 5.7, 11.4 µg/L; 10.6375, 21.275, 42.55, 85.11, 170.2 µg/L	adult	96 h	immersion	the whole body	24 h impaired feeding	oxidative stress	[37]
*Annelida*	*Paracyclopina nana*	0.1, 1, 10 µg/L	12 hph nauplii	14 d	immersion	the whole body	developmental toxicity, increased lipid production	oxidative stress, activation of MAPK signaling cascade (ERK and JNK), de novo lipogenesis	[68]
*Mollusca*	*Ruditapes philippinarum*	5 µg/L	adult	15 d	immersion	digestive glands	--	suppression subtractive hybridization (SSH)-transcriptome	[69]
		5 µg/L	BBL = 4.06 ± 0.42 cm, BH = 2.53 ± 0.73 cm, BW = 8.32 ± 0.98 g	15 d	immersion	gill, digestive gland	impaired morphology, developmental toxicity	DNA damage	[69]
		6.25 µM, 12.5 µM, 25 µM, 50 µM, 100 µM	BL = 3.49 ± 0.26 cm	12 h	immersion	hemocytes	immunotoxicity	oxidative stress, MAPKs (reduced p-ERK expression, disturbed p38 expression)	[70]
	*Mytilus edulis*	0.1, 1.0, 10 µg/L	adult (BL = 3.5–5.0 cm)	21 d	immersion	hemocytes	altered energy metabolism	target metabolism (impaired TCA cycle and glycolysis)	[1]
		0.1, 1, 10 µg/L	BL = 3.5–5.0 cm	21 d	immersion	hemocytes	impaired morphology, immunotoxicity	oxidative stress	[46]
		0.23 µg/L	adult	3 w	immersion	digestive glands	--	proteomics (non-high-throughput)	[71]
		5 ppb	adult	3 w	immersion	gill	--	DNA damage	[72]
	*Mytilus galloprovincialis*	10 µg/L	BW = 12.9 ± 1.4 g, BL = 5.7 ± 0.74 cm	26 d	immersion	digestive gland, gills, gonad	impaired morphology, bioaccumulation	oxidative stress	[42]
		1, 10 µg/L	adult, BL = 5.5–6.0 cm	30 d	immersion	gonad	impaired metabolism	impaired metabolism (metabolomics)	[73]
	*Mytilus coruscus*	0.1, 1, 10 µg/L		21 d	immersion	hemocytes	immunotoxicity	oxidative stress	[25]
	*Crassostrea gigas*	100, 200, 400, 800, 1600 µg/L	egg-larvae	96 h	immersion	the whole body	developmental toxicity	DNA damage	[74]
*Chordata*	*Oncorhynchus mykiss* gonadal RTG-2 cells	6, 12.5, 25 µM	--	6 and 24 h	immersion	gonadal RTG-2 cells (No. CCL-55)	impaired morphology, reproductive toxicity	apoptosis, excessive produced Ca^2+^	[45]
		6, 12.5, 25 µM	--	6 and 24 h	immersion	gonadal RTG-2 cells (No. CCL-55)	reproductive toxicity	apoptosis, oxidative stress activates the Nrf2-mediated antioxidant response and P38 MAPK pathway	[75]
	*Oncorhynchus mykiss*	42.35 ng/g, 372.71 ng/g	juvenile, wet BW = 10–15 g, BL = 9.4 ± 3.7 cm	21 d	food intake	the head kidney	--	lipid peroxidation, PXR-mediated detoxification, Nrf2-mediated antioxidation system	[76]
	*Cynoglossus semilaevis* Gunther	5, 500, 50,000 ng/L	BL = 3.0 ± 0.5 cm, BW = 2.4 ± 0.5 g	15 d	immersion	liver	--	oxidative stress	[77]
	*Gadus macrocephalus* Tilesius	5, 500, 50,000 ng/L	BL = 45 ± 6.8 cm, BW = 1225 ± 395 g	96 h	immersion	mussel, blood	--	oxidative stress, DNA damage	[78]
	*Oryzias melastigma*	--	adult	21 d	food intake	liver, muscle	bioaccumulation	sex specific effects on apoptosis and heat shock protein expression	[79]
		--	adult	21 d	food intake		intergenerational transmission, bioaccumulation	transmission through lipids	[80]
	*Psetta maxima*	0.3, 0.75, 1.5, 2.5, 3.125, 6.125, 12.5, 25, 30, 100, 150, 200 µg/L	72 h egg	6 d	immersion	the whole body	developmental toxicity	--	[81]

h: hours; d: days; w: weeks; m: months; BL: the average body length; BW: the average body weight; BH: the average height of the shells.

### 2.3. BDE-47-Induced Impaired Motility

BDE-47 can significantly inhibit the movement of planktonic organisms. The EC_50_ for the inhibitory effect on the movement of *Brachionus plicatilis* within 24 h is reported to be 9.695 mg/L [38]~9.7 mg/L [32]. The 24 h LOECSI (the lowest observed effect concentration for swimming inhibition) for *Brachionus plicatilis* obtained through SSA testing is 2.0 mg/L [48].

Some studies suggest that, compared to traditional methods of assessing seawater quality using parameters such as population growth and measuring photosynthesis, the use of motion inhibition parameters (MOT, VCL, and VAP) is more sensitive and effective for seawater quality assessment [48]. They utilized the motile marine green algae *Platymonas subcordiformis* to count cells that remained in motion (VCL ≥ 25 µm/s) after 2 h of exposure to gradient concentrations. The statistical parameters, including MOT, VCL, and VAP, are defined as follows. MOT is the proportion of motile cells in the field of view. VCL is the time-averaged velocity of a cell along its actual curvilinear path. VAP is the time-averaged velocity of a cell along an average path, constructed using a roaming average of cell position from one-sixth of the video’s frame rate, respectively. MOT is already reduced at 1 µg/L BDE-47 exposure. Subsequently, in a concentration-dependent manner, it further decreases to 0 when the concentration reaches as high as 32 µg/L. Both VCL and VAP also significantly decrease at all exposure concentrations, with EC_50_ values of 5.46, 6.83, and 6.69 µg/L, respectively.

### 2.4. Photosynthesis Toxicity

As the primary component of the marine food web, phytoplankton holds crucial significance in the study of bioaccumulation, transformation, and toxicity of marine pollutants. The toxic effects of BDE-47 on phytoplankton vary depending on the species. To date, research on the impact of BDE-47 on marine microalgae has encompassed 11 species across four phyla: *Chlorophyta*, *Bacillariophyta*, *Chrysophyta*, and *Pyrrophyta*.

BDE-47 can lead to the redistribution of pigments within algae cells. Liu et al. found a significant reduction in chlorophyll A content in *Phaeodactylum tricornutum* exposed to 0.8 and 4 mg/L BDE-47, reaching only 79.0% and 72.8% of the control group, respectively (*p* < 0.05) [44]. In contrast, Zhang et al. discovered a significant increase in chlorophyll A content after exposure to 600 µg/L BDE-47 for 24 to 96 h [40]. Zhao et al. observed a significant increase in TC/chl a and chl c/chl A ratios in two algae, *D. salina* and *A. minutum*, exposed to 0.074, 0.365, and 0.697 mg/L BDE-47 for 120 h [39]. The increase in TC/chl a indicates a defense mechanism against oxidative stress, as some major components of carotenoids, such as β-carotene and lutein, act as antioxidants in plants.

Specifically, in terms of toxicity to primary producers, BDE-47 can induce damage to the photosynthetic process of microalgae by stimulating the excessive production of reactive oxygen species (ROS) [44]. In a study conducted by Zhao et al. [43], accompanied by oxidative stress, cell death, and programmed cell death (PCD), 96 h exposure of *Chlorella* sp. to BDE-47 resulted in significantly lower values for all photosynthetic (PS II) parameters compared to the control group. Additionally, under BDE-47 stress, the production of reactive oxygen species (ROS) was found to be directly proportional to the toxicity of photosynthesis [44].

Under BDE-47 stress, photosynthesis in algae is significantly inhibited. In the case of *Phaeodactylum tricornutum* exposed to 600 µg/L BDE-47, the inhibition rates for Fv/Fm, rETR.max, and ΦPSII can reach 55.2%, 69.46%, and 50%, respectively [40]. Liu et al. observed a significant increase in non-photochemical quenching coefficients in the diatom *Skeletonema costatum* exposed to 0.8 and 4 mg/L BDE-47. Additionally, oxygen evolution rates, Fv/Fm values, and rETR were significantly reduced under BDE-47 exposure [44]. In experiments with *D. salina* and *A. minutum* exposed to a series of concentrations of BDE-47 for 120 h, a significant decrease in rETR.max was found, making it the most affected parameter among all measured parameters [39]. rETR.max represents the maximum electron transfer rate in the light reactions. In the chloroplasts, reactive oxygen species (ROS) influence the conversion of O_2_ to O_2_^−^, leading to a reduction in the electron transfer rate. A decrease in rETR.max may result in reduced energy transported to the dark reaction sites, ultimately inhibiting the rate of carbon fixation and damaging photosynthesis. Liu et al. further explored the mechanism at the transcriptome level, indicating that the down-regulation of genes related to photosynthesis, particularly those encoding fucoxanthin chlorophyll a/c binding proteins and involved in porphyrin and chlorophyll synthesis and carbon fixation, contributed to these negative effects [44].

### 2.5. Immunotoxicity

BDE-47 induces lipid peroxidation and membrane damage through the excessive production of reactive oxygen species (ROS), thereby disrupting the structure of genetic material and cellular ultrastructure, leading to impaired immune function in organisms [82,83]. Additionally, the immunotoxicity may be partly attributed to alterations in the MAPK signaling pathway.

Zhou et al. conducted an exposure study using hemocytes of the Philippine clam (*Ruditapes philippinarum*) exposed to five concentrations (6.25 µM, 12.5 µM, 25 µM, 50 µM, 100 µM) of BDE-47 within 12 h. The study revealed that BDE-47 exposure reduced hemocyte viability, decreased the granulocyte ratio, hindered hemocyte phagocytosis and bacterial degradation activity, elevated ROS levels, increased lysosomal membrane permeability (LMP), significantly altered the expression of the immunologically important enzyme SOD, and decreased the phosphorylation levels of ERK and p38 in the MARK pathway [70].

After exposure to 10 µg/L for 21 days, the immune-related enzyme ACP in mussels *M. coruscus* showed a significant increase in activity, indicating the activation of the immune system [25]. Jiang et al. conducted a community structure analysis of hemocytes from blue mussels (*Mytilus edulis*) exposed to pollutants using flow cytometry. They found significant changes in parameters closely related to hemocyte community structure and immune function in the exposed group [46]. With increasing concentration, the mortality of blue mussels increased, leading to a decrease in THC content in their hemocytes. However, with prolonged exposure time, the number of hemocytes in the exposed group increased, possibly due to a compensatory immune function in response to long-term stress. Changes in lysosome structure were observed under exposure, NRRT in cells significantly decreased, and granules in granulocytes formed vesicles under electron microscopy during exposure. The cell nucleus also underwent morphological changes, showing chromatin condensation, which could affect the immune function of hemocytes at the expression level [46]. The process of phagocytosis corresponds to the membrane characteristics of hemocytes, is an essential component of the immune response in invertebrates, and is considered a biomarker reflecting environmental pollution [46]. After long-term exposure for 21 days, the phagocytic activity of blue mussels (*Mytilus edulis*) significantly decreased [46]. With increasing exposure time and concentration, ROS and MDA levels significantly increased, showing a strong correlation with the aforementioned indicators [46].

### 2.6. Liver Toxicity Induced by BDE-47

The liver stands out as a pivotal target for both the accumulation and toxic impacts of BDE-47 within the organisms. Liu et al. discovered that rainbow trout fry (10–15 g wet weight, 9.4 ± 3.7 cm) exposed through a 21 day dietary intake (concentrations in the feed equivalent to 42.35 ng/g and 372.71 ng/g for BDE-47) exhibited the highest bioaccumulation of BDE-47 in the liver, followed by the head-kidney, muscle, stomach, and spleen [76]. Chi et al. performed a 15 day exposure experiment on the economically valuable marine fish species, *Cynoglossus semilaevis* Gunther, in three concentrations (5, 500, and 50,000 ng/L). In the liver, they observed no significant changes in various antioxidant enzyme activities, ER content, and EROD activity under environmental concentration BDE-47 exposure. However, in the medium and high concentration groups (500 ng/L and 50,000 ng/L), there were significant differences compared to the control group, indicating oxidative stress damage to the liver of the fish [77].

### 2.7. Developmental Toxicity

BDE-47 significantly impacts the early development of marine organisms, showing increased sensitivity in various species during their initial life stages.

Exposure to higher concentrations (i.e., 19.53, 97.65, and 130.2 µg/L) of BDE-47 caused a significant increase in embryo mortality in the marine flatfish turbot *Psetta maxima* after just 48 h. Surviving organisms experienced a notable decrease in hatching success, as well as malformations in embryos, pericardial edema, and skeletal deformations in larvae at 96 h post-hatching (hph). The NOEC and LOEC values for embryos protected by the chorion were higher (2.03 and 4.07 µg/L) compared to the naked larvae (0.49 and 1.63 µg/L) [81]. Xie et al. exposed Pacific oyster *Crassostrea gigas* embryos for 24 to 96 h until they developed into the D-shaped larval stage. At 24 h, a significant increase in abnormal D-shaped larvae was observed at a concentration of 72.5 µg/L. Furthermore, protruding mantle, convex hinge, and incomplete shell were observed at 96 h. The EC_50_ was calculated as 203.3 (162.0–271.5) µg/L, and the LOEC was determined as 72.5 µg/L.

Most studies to date indicate that under the stress of BDE-47, many marine organisms undergo a shortened developmental cycle. For instance, experiments using post-hatching copepods *Paracyclopina nana* over a period of 14 days revealed a significant reduction in developmental time at 10 µg/L of BDE-47 (*p* < 0.05) [68]. Additionally, concentrations of BDE-47 at 0.008 mg/L and above significantly shortened the time to reach the time-to-inflection point of growth curve (Tp) in the copepod species *B. plicatilis* [66]. However, some studies have suggested that exposure to 120 µg/L BDE-47 resulted in delayed development in the intertidal *Tigriopus japonicas*, slowing the transition from nauplius larvae to copepodites and adult stages [67].

### 2.8. Reproductive Toxicity

BDE-47 demonstrates notable toxicity in the population growth and reproduction of planktonic organisms, displaying a concentration-dependent pattern [32,58,59,60,63,66]. At 120 µg/L BDE-47 exposure, the reproductive rate of *Tigriopus japonicas* significantly decreased by 50% (*p* < 0.001) [67].

BDE-47 has been found to expedite the developmental stages of rotifers, implying that exposure accelerates the sexual maturation of these organisms. This appears to induce a shift in the reproductive strategy of rotifers. Ovaries appear to be one of the crucial targets for the toxic effects of BDE-47. Severe damage to the ovaries has been observed under BDE-47 exposure. After 24 h of exposure to 0.008 mg/L BDE-47, ovarian follicles in the rotifer appeared wrinkled and lost their characteristic appearance, with a slight deformation of the cell nucleus and denser chromatin spots. Compared to the control group, there was a decrease in lipid electron density. At a higher concentration (0.08 mg/L), the follicles underwent complete deformation, with a significant deformation of the cell nucleus and denser chromatin spots. Additionally, lipid electron density was significantly reduced compared to the control group.

BDE-47 induces mitochondrial damage and cell apoptosis in ovarian cells by stimulating the production of ROS and Ca^2+^. In the exposed ovaries of *Brachionus plicatilis*, Tang et al. observed a reduction in mitochondrial membrane potential (MMP) and increased fluorescence intensity, indicating apoptosis in ovarian cells. Furthermore, significant ultrastructural damage to the mitochondria in the ovaries was revealed using transmission electron microscopy [47]. Two studies have highlighted the pronounced toxic effects of BDE-47 exposure on rainbow trout gonad (RTG-2) cells. Apoptosis of gonadal cells was initiated at concentrations ranging from 6 µM to 25 µM, while exposure to 25 µM BDE-47 resulted in mitochondrial swelling and partial dissolution [45,75]. In female *Brachionus plicatilis* ovaries exposed to 0.05 mg/L, 0.1 mg/L, and 0.2 mg/L BDE-47, a substantial increase in ROS and Ca^2+^ levels was observed, surpassing those in other organs (such as the gland and stomach). This elevation positively correlated with exposure concentration, suggesting it could be a significant mechanism contributing to reproductive toxicity [65].

In 2005, Haraguchi et al. found higher concentrations of BDE-47 in calf blubber than in lactating females in blubber samples from stranded killer whales (*Orcinus orca*) using gas chromatography-mass spectrometry (GC-MS) [84]. A more recent study by Durante et al. is a more systematic description of the relationship between fetal and maternal BDE-47 ratios in marine mammal cetaceans [85]. In addition to this, the researchers looked more deeply into the maternal transfer of BDE-47 in marine animals and found that lipid mobilization can facilitate this process [86]. In a study conducted by van de Merwe et al., female fishes *Oryzias melastigma* were subjected to dietary exposure by embedding BDE-47 in brine shrimp. During the 21 day exposure period, each 2 month old (pre-breeding) fish ingested 1.3 ± 0.2 micrograms of dietary BDE-47 per day. At the end of the exposure, although there was no significant difference in the final concentrations of BDE-47 between males and females, the concentration in females was notably lower after egg laying. Notably, the accumulated concentration of BDE-47 in the eggs reached 25 ng/egg on the 18th day, highlighting the transfer of BDE-47 from mothers to offspring, a process intricately associated with lipid mobilization [80].

## 3. Molecular Mechanisms of BDE-47-Induced Toxicity

The molecular mechanisms underlying the toxicity of BDE-47 in marine organisms seem to originate from the excessive production of ROS and Ca^2+^, and its molecular mechanisms involve the activation of oxidative stress pathways, detoxification systems, metabolic processes, cellular apoptosis, and the cascading activation of MAPK (Mitogen-Activated Protein Kinase).

### 3.1. The Activation of Oxidative Stress Pathways

Nearly all studies have reported a concentration-dependent overproduction of Reactive Oxygen Species (ROS) in organisms across various trophic levels following exposure to BDE-47 (e.g., [25,43,44,65,67,75,76]). Concomitant with the overproduction of ROS, alterations in the activities of redox system-related enzymes were observed. For instance, in the presence of a high concentration (600 µg/L) of BDE, *Skeletonema costatum* exhibited varying degrees of increase in Superoxide Dismutase (SOD) and Malondialdehyde (MDA) concentrations [40]. Within 24 h, the exposure of *P. nana* larvae to BDE-47 (0, 0.1, 1, and 10 µg/L) led to a significant elevation in intracellular ROS levels (*p* < 0.05). In comparison to the control group, the activities of Glutathione S-Transferase (GST) and Glutathione Peroxidase (GPx) significantly increased at all concentrations (*p* < 0.05) [68]. Jian et al. [41] exposed newly hatched *P. nana* for 2 h and observed that ROS concentrations and MDA contents increased consistently with rising BDE-47 concentrations. The MDA content showed approximately 20%, 86%, and 108% increases in the moderate (0.08 mg/L) and high (0.8 mg/L) concentration groups. This pattern aligned with the elevated ROS concentrations of 103% and 105% in the same treated group. The GSH/GSSG value experienced a significant reduction (*p* < 0.05) by 24% and 25% in the 0.08 mg/L and 0.8 mg/L groups, respectively, indicating oxidative stress. This observation is consistent with previous research [48]. Specifically, in female zooplankton, the ovaries seem to be a significant site for the substantial generation of ROS and oxidative stress [65]. Similarly, a reduction in GST and GSH levels, coupled with an elevation in SOD and MDA content, was observed in blue mussels exposed to environmentally relevant concentrations of BDE-47 [42].

The antioxidant mechanisms vary across different organisms. In a study by Zhao et al., the antioxidant system response in *Alexandrium minutum* and *Dunaliella salina* exposed to BDE-47 (0.074, 0.365, 0.697 mg/L) was examined. Exposure led to an increase in the production of reactive oxygen species (ROS), with distinct variations observed in the activities of the four key enzymes involved in ROS clearance. In *Dunaliella salina*, GPX activities showed reductions of 16%, 50%, and 53% in the high, medium, and low BDE-47 treatments, respectively (*p* = 0.271, *p* = 0.002, and *p* = 0.003). Conversely, GR activity exhibited increments of 35%, 118%, and 94% in the high, medium, and low BDE-47 treatments, respectively (*p* = 0.001, *p* = 0.001, and *p* < 0.001). In *Alexandrium minutum*, both GPX and GR activities increased. GPX activity rose by 68%, 71%, and 126% in the high, medium, and low BDE-47 treatments, respectively (*p* = 0.139, *p* < 0.001, and *p* = 0.002). Additionally, GR activity increased by 348%, 393%, and 74% in the high, medium, and low BDE-47 treatments, respectively (*p* < 0.001, *p* < 0.001, and *p* = 0.133).

The cell membrane emerges as a principal site for the generation of reactive oxygen species (ROS) as well, playing a pivotal role as essential signaling molecules in orchestrating the programmed cell death (PCD) process [56]. Exposure of blue mussels to 0.1~10 µg/L BDE-47 resulted in a time- and dose-dependent overproduction of reactive oxygen species (ROS) in hemocytes. Based on observed indicators, such as hemocyte ultrastructural damage, changes in cell community proportions, and lysosomal damage, among others, this study suggests that BDE-47 can induce the excessive generation of ROS in hemocytes. This induction leads to membrane damage, ultimately causing structural and functional alterations in hemocytes across different biological spectra.

Nrf2-mediated antioxidant system is a confirmed oxidative stress pathway. In juvenile rainbow trout exposed to dietary concentrations of 42.35 ng/g and 372.71 ng/g BDE-47 for 21 days, a significant increase in MDA content was observed in the head kidney, indicating oxidative stress. During the early stages of BDE-47 exposure, the expression of Nrf2, SOD, GST, and their respective enzymes was increased, activating the Nrf2-mediated antioxidant system. However, with prolonged exposure, gene expression continued to rise, while enzyme activity was inhibited, leading to the disruption of the antioxidant system [76]. A similar experiment using rainbow trout gonadal cells also revealed a substantial increase in the mRNA levels of nuclear factor erythroid 2-related factor 2 (Nrf2) and its downstream genes under BDE-47 exposure [75].

### 3.2. The Activation of Detoxification System

PXR, a nuclear receptor sensitive to both internal and external compounds, plays a regulatory role in the expression of detoxifying enzymes in both Phase I and Phase II. Under BDE-47 exposure, there is a significant negative correlation between the activation of PXR and BDE-47 concentration (*p* < 0.01) as well as MDA content (*p* < 0.05). The activation of PXR under BDE-47 exposure manifests a “low promotion, high inhibition” effect. Liu et al. carried out a dietary exposure study on rainbow trout fry (10–15 g wet weight, 9.4 ± 3.7 cm) using feed containing BDE-47 at concentrations of 42.35 ng/g and 372.71 ng/g for 21 days, based on previous research. They observed that the expression of *PXR* and *CYP3A* genes was significantly higher than the control group in the early exposure period (7 days and 14 days) and subsequently decreased by day 21. Simultaneously, the activities of detoxifying enzymes, specifically CYP3A in phase I and GST in phase II, were measured and showed a similar trend to the gene expression. The authors concluded that BDE-47 could initially enhance the expression of gene *PXR*, *CYP3A*, and *GST*, as well as the enzymatic activity of detoxifying enzymes in phases I and II. However, with prolonged exposure, the metabolic detoxification capacity of BDE-47 declined, and significant inhibition was observed.

Under exposure to BDE-47, the expression levels of TJ-catalase and Tj-Mn SOD in *Tigriopus japonicas* were regulated by BDE-47. Significant expression (*p* < 0.05) was observed in the gene *Tj-GST-sigma*, and the expression of detoxification-related genes, namely *Tj-CYP3027C2* and *Tj-CYP3024A2*, was significantly higher than that in the control group (*p* < 0.05) [67].

In marine mammal-related research, cellular-level studies are mainly conducted by establishing cell lines. Rajput et al. found that BDE-47 was able to enhance the expression of cytokines such as IL-1β, IL-8 and other cytokines, which had an effect on cellular immune response using molecular biology methods such as real-time fluorescence quantitative PCR (RT-PCR), protein blotting and immunofluorescence assay [87].Ying et al.’s research found that BDE-47 was able to reduce the expression level of prostaglandin E-2 receptor EP4 and inhibit the expression level of anti-inflammatory cytokines in the dolphin fibroblast cell line repertoire [83].

### 3.3. DNA Damage and Apoptosis

BDE-47 has been shown to induce damage to DNA in marine organisms exposed to it [82]. In a study conducted by Xie et al., the comet assay demonstrated a direct correlation between the extent of DNA damage and the rate of abnormalities in *Crassostrea gigas* D-shaped larvae after a 16 h exposure to BDE-47. The percentage of tail DNA increased significantly with escalating concentrations of BDE-47 [74]. Meanwhile, in blue mussels exposed to sub-lethal concentrations (5 ppb) for three weeks, gill cells exhibited distinctive features such as binucleation, fragmented apoptotic cells, and nuclear budding, indicative of potential DNA damage [72].

#### 3.3.1. ROS Mediated Mechanism

Reactive oxygen species (ROS)-mediated programmed cell death (PCD) stands out as a crucial and conserved toxic mechanism activated by polybrominated diphenyl ethers (PBDEs) across different species. For instance, studies such as that by Zhao et al. have identified ROS-dependent PCD in microalgae exposed to BDE-47 treatment [43]. Organisms exposed to BDE-47 exhibit time- and dose-dependent oxidative stress and DNA damage [78]. This exposure results in the upregulation of apoptosis-related genes, such as *p53* and *Rb* [67], subsequently triggering DNA repair mechanisms, as evidenced by the upregulation of the expression of the cell proliferation gene *PCNA*.

Previous studies have shown that significant amounts of reactive oxygen species (ROS) are generated in the chloroplast and cell membrane. Under BDE-47-induced stress, ROS production is notably increased in the cell membrane and chloroplast, serving as crucial signaling molecules that trigger programmed cell death (PCD) in the model diatom *Thalassiosira pseudonana*. Compared to the control group, the experimental group exhibited elevated levels of DNA fragmentation, PS externalization rate, and cysteine aspartate-specific protease (caspase) activity. Moreover, the mRNA expression of PCD-associated genes like *HSP70*, *TpMC2*, *DAP3*, and *TpDSP1* was upregulated. Pearson correlation analysis demonstrated a significant correlation (*p* < 0.01) between ROS levels and three key biochemical indicators of PCD:ROS positivity rate, dead cell rate, and the levels of the three PCD biochemical markers [56].

Furthermore, mitochondria emerge as a pivotal site for the generation of reactive oxygen species (ROS). Under a series of exposures to BDE-47 in the *Brachionus plicatilis*, a decrease in mitochondrial membrane potential and an enhancement in fluorescence intensity were observed [47]. Moreover, exposure to BDE-47 induced an increased ratio of the pro-apoptotic protein Bax to the anti-apoptotic protein Bcl-2 (Bax/Bcl-2), signifying the onset of cellular apoptosis. Notably, mitochondrial integrity was disrupted by BDE exposure, leading to the first observed instances of mitochondrial autophagy in rotifer cells exposed to concentrations of 0.08 and 0.8 mg/L BDE-47. Furthermore, the study identified a concentration-dependent escalation in the activity of critical components in the mitochondrial pathway, namely caspase-9 and caspase-3, accompanied by a substantial production of ROS. These ROS, in turn, exacerbated damage to the mitochondrial pathway, triggering apoptosis and initiating a detrimental cycle.

Purine and pyrimidine metabolism dysregulation may play a crucial role in reactive oxygen species (ROS)-mediated DNA damage and cellular apoptosis. In a recent metabolome analysis conducted by Cao et al., *Brachionus plicatilis* was exposed to BDE-47 for 24 h. Under the influence of BDE-47 stress, an excessive generation of reactive oxygen species (ROS) occurred, subsequently resulting in elevated levels of the DNA damage marker 8-OHdG, increased expression of the p53 protein, and an elevated Bax/Bcl-2 ratio. Additionally, noteworthy changes were observed in the purine and pyrimidine content in the treated group, accompanied by alterations in the activities of two key enzymes involved in purine and pyrimidine metabolism: a downregulation of glutamine synthetase (GS) protein expression and an increase in xanthine oxidase (XOD) activity [61].

Currently, due to experimental techniques and design problems, most studies have been limited to lower marine organisms and fish species, and fewer studies have been conducted on the toxicity of higher organisms, especially mammals. However, due to the long-term presence of BDE-47 in the environment and its ability to biomagnify through the food web, BDE-47 is detected at high concentrations in marine mammals, especially cetaceans. The study of the molecular mechanisms of BDE-47 toxicity in marine mammals is of critical importance. However, conducting toxicity studies in marine mammals is challenging due to ethical, regulatory, and technical constraints. Liu et al. established a pan-tropical spotted dolphin (*Stenella attenuata*) skin fibroblast cell line (PSD-LWHT) and pygmy killer whale skin fibroblast cell lines (PKW-LWHT) and utilized the in vitro cell lines to quantitatively assess the cytotoxicity, genotoxicity, and immunotoxicity of BDE-47 and its major transformed product, 6-OH-BDE-47, as well as the specific molecular mechanisms [82,88]. It was found that exposure to 1 µg/mL BDE-47 increased reactive oxygen species (ROS) production, while superoxide anion, hydroxyl radical and inducible nitric oxide increased in a dose-dependent manner. At 0.5–1.0 µg/mL, BDE-47 significantly decreased the mitochondrial membrane potential, which in turn led to dysregulation of oxidative stress as well as alteration of mitochondrial and cell membrane structure and activity in fibroblasts. Whereas 6-OH-BDE-47 upregulated p21 gene expression and inhibited RB1 gene phosphorylation by interfering with the formation of CDK4, CDK6/cyclin D complex and CDK2/cyclin E complex. The cell cycle analysis by flow cytometry and PI staining showed that the DNA replication was inhibited, and the PKW-LWHT cell cycle stagnated in the G0/G1 phase, thus inhibiting the proliferation of PKW-LWHT cells.

#### 3.3.2. Ca^2+^ Mediated Mechanism

Ca^2+^ plays a fundamental role as a pivotal messenger mediating stress responses in metazoans and microalgal cells. There is an interplay between Ca^2+^ and ROS, and the levels of ROS are, to some extent, influenced by cellular Ca^2+^ concentration. The Ca^2+^ content in *Chlorella* sp. cells is found to increase with the rise of ROS and decrease with the addition of the ROS scavenger NAC [43]. Under exposure to BDE-47, a significant increase in Ca^2+^ fluorescence intensity was observed in female rotifers, particularly in the mastax, stomach, and ovaries, with the strongest fluorescence detected in the ovaries. The fluorescence levels in different regions showed a positive correlation with the exposure concentration, indicating significant increases in Ca^2+^ at 0.1 mg/L BDE-47 (*p* < 0.01) and 0.2 mg/L BDE-47 (*p* < 0.001) compared to the control. Additionally, the expression of *calmodulin* (*CaM*) mRNA exhibited dose-dependent enhancement. In experiments involving rainbow trout gonadal cells, it was discovered that exposure to BDE-47 induces apoptosis in RTG-2 cells through the mitochondria, endoplasmic reticulum, and death receptor apoptosis pathways mediated by ROS and Ca^2+^ [65].

### 3.4. Alternated Energy Metabolism and Compromised Metabolic Functions

Sub-lethal concentrations of BDE-47 can inhibit the feeding behavior of nematodes, primarily attributed to disruptions in energy metabolism and neural transmission [62]. Exposure to sub-lethal concentrations (31.25, 125, and 500 µg/L) for 24 h resulted in a significant decrease in the feeding and filtering rates of *Brachionus plicatilis*, accompanied by the accumulation of BDE-47 within the organisms. Furthermore, the activities of amylase (AMS) and protease were affected, indicating that BDE-47 impairs intake and digestive efficiency.

BDE-47 may stimulate ROS production, thereby affecting the tricarboxylic acid (TCA) cycle and glycolysis in organisms, altering their energy metabolism. In a study by Jiang et al. [1], adult blue mussels (*Mytilus edulis*, shell length 3.5–5.0 cm) were exposed to a BDE-47 solution for 21 days. Subsequent analysis of their hemocytes involved the measurement of enzymes and genes related to the TCA cycle and glycolysis, combined with targeted metabolomics. Results revealed that under low BDE-47 exposure (0.1 µg/L), the energy supply pattern in mussels shifted from normal aerobic respiration to anaerobic mode, allowing for rapid energy acquisition and enhanced nutritional accumulation. At low concentrations, peroxidation inhibited the activity of key enzymes in the TCA cycle, including IDH, SDH, and MDH. Enzyme activity and gene expression associated with glycolysis increased, coupled with a decrease in LDH activity and overexpression of LDHA, indicating that enhanced glycolysis promoted anaerobic respiration in hemocytes. The activation of the AMPK-Hif-1a signaling pathway induced the expression of glut1, further activating glycolysis and anaerobic respiration. As the BDE-47 concentration increased, the primary energy supply mode shifted back to aerobic respiration, providing increased energy for cellular metabolism. BDE-47 exposure in nematodes also induced damage to the mitochondrial morphology at the cilia root of the digestive tract. The activities of two key enzymes involved in the tricarboxylic acid (TCA) cycle, citrate synthase (CS), and isocitrate dehydrogenase (IDH), significantly decreased, leading to mitochondrial dysfunction. Additionally, the activity of ATPase, which catalyzes ATP hydrolysis decreased with increasing concentration of BDE-47, implying a delay in nematode ATP kinetics and consequent disruption in ciliary movement for food ingestion. Moreover, a significant decrease in acetylcholinesterase activity was observed, indicating that neural transmission related to food intake and digestion in BDE-47-exposed nematodes was also impeded.

In recent years, with the rapid development of high-throughput sequencing technology and big data processing capability, applying omics technology has gradually become a hot topic in toxicology, and there have been omics studies on the mechanism of metabolic effects of BDE-47 on marine organisms. Due to the relative simplicity of the morphological structure of the model organism, its more basic function, and the better understanding of its gene expression, there are more omics studies on rotifers. The transcriptomic analysis conducted by Yang et al. [89] on marine rotifer (*Brachionus plicatilis*) acutely exposed to BDE-47 revealed significant enrichment in metabolism-related differentially expressed genes (DEGs), extracellular matrix (ECM)-receptor interaction-related DEGs providing structural support to cells, and lysosomal-related DEGs. Another metabolomics study [61] on marine rotifers (*Brachionus plicatilis*) exposed to BDE-47 identified three major sensitive pathways in response to BDE-47: aminoacyl-tRNA synthesis, the biosynthesis of valine, leucine, and isoleucine, and arginine biosynthesis. These pathways were significantly affected under exposure, leading to a decrease in the levels of the amino acid pool. Additionally, both pyrimidine and purine metabolism pathways were influenced, resulting in disruptions in the synthesis and degradation of nucleotides within the organism. As the application of omics became more sophisticated, researchers gradually turned to higher organisms. Currently, the study of omics has been conducted on different levels of organisms to reveal the similarities and differences in the metabolic effects of BDE-47 on different levels of organisms. A comprehensive analysis of the whole transcriptome was conducted by Zhao et al. on the diatom *Thalassiosira pseudonana* following exposure to BDE-47 [55]. The study revealed a significant enrichment of differentially expressed genes associated with oxidative stress, inhibition of light reactions, and the upregulation of genes related to various aspects of carbon metabolism such as the Calvin cycle, TCA cycle, glyco56lysis, fatty acid synthesis, and triglyceride synthesis. Additionally, genes involved in DNA damage, including DNA repair, cell cycle arrest, and programmed cell death, were also found to be upregulated. These molecular mechanisms collectively contribute to the inhibition of cell division and population growth in diatom populations exposed to BDE-47. For the lower zooplankton, a study of the copepod *Tigriopus japonicus* showed an increase in the transcript levels of genes related to de novo lipogenesis (DNL) after 24 h of exposure to 2.5 µg/L BDE-47 and found that the metabolic intermediates palmitic acid was significantly increased on days 1 and 4, and docosahexaenoic acid and arachidonic acid were down-regulated on days 1 and 4 [90]. However, more studies have focused on marine invertebrates. In 2012, Zhang et al. found that different concentrations (0.1, 1 and 10 µg/L) of BDE-47 affected molluscan sex steroid signaling mechanisms in the ctenophore scallop, as evidenced by different increases in the mRNA transcripts of estrogen receptor (ER) and Vitellogenin(VTG) [91]. Shi et al. exposed clams to 5 µg/L BDE-47 for 3 days, and then performed high-throughput sequencing to detect 17,625 differentially expressed genes (DEGs) in clams [92]. DEGs are enriched for metabolic functional pathways, such as detoxification, antioxidant defense, immune response, and apoptosis. In 2014, metabolomics was utilized to study the sex-specific metabolic response of BDE-47 on the gonads of the mussel *Mytilus galloprovincialis* [73], which revealed that a low concentration of BDE-47 (1 µg/L) was able to elicit osmotic stress in the male mussel gonad, whereas high concentrations (10 µg/L) elicited disruption of osmoregulation and increased energy demand, in contrast to low concentrations of BDE47 in female mussels that elicited reductions in energy regeneration systems, such as ATP and arginine, whereas increased fatty acid oxidation was observed at high concentrations. Geng et al. [93] conducted a joint analysis of transcriptomics and metabolomics on blue mussels acutely exposed to BDE-47. The results indicated a high correlation between the metabolomic data and transcriptomic data, suggesting that changes in differentially expressed metabolites (DEMs) may be regulated by corresponding DEGs. Under individual exposure to BDE-47, the energy metabolism pathway of blue mussels was disrupted, with both DEMs and DEGs significantly enriched in the PI3K-Akt signaling pathway, and notable changes occurred in inflammation-related DEGs. Notably, in 2023, Ding et al. shifted their study to the neurotoxicity of BDE-47 in sea cucumber and found significant changes in metabolites, such as steroid hormone homeostasis, nucleotide metabolism, energy metabolism, neurotransmitter levels, and neuroprotection, at different exposure concentrations (0.1, 1, and 10 µg/L) by metabolome [94]. For fish, Yu et al. found that BDE-47 sex-specifically affects the hepatic transcriptional profile of marine medaka (*Oryzias melastigma*) by transcriptomics, and may activate phosphatidylinositol-3-kinase and mitogen-activated protein kinase, proteins that play important roles in cell growth, proliferation, and survival, only in males [95].

In summary, researchers have gained a more systematic and specific molecular level knowledge of the metabolic changes in BDE-47 on different marine organisms through the transcriptome, proteome, and metabolome, and found that metabolic toxicity of BDE-47 exhibits species specificity in marine organisms, which may be attributed to different strategies in response to external stimuli. For instance, studies have indicated that *Eurytemora pacifica* exhibits heightened increased feeding rates and ammonia excretion at moderate concentrations (1.425 µg/L), while oxygen consumption is significantly inhibited at high concentrations (5.70 and 11.40 µg/L). On the other hand, *Tigriopus japonicas* experienced suppressed feeding rates at high BDE-47 concentration (170.20 µg/L), but enhanced oxygen consumption at moderate levels (21.28 µg/L). Across different concentrations, BDE-47 consistently triggers ammonia excretion in *Tigriopus japonicas* [37].

### 3.5. Activation of the MAPK Signaling Cascade

MAPKs (Mitogen-Activated Protein Kinases) constitute a superfamily of protein Ser/Thr-kinases that regulate physiological functions by activating a cascade of protein kinases in the cytoplasm or by translocating into the nucleus to modulate gene expression. The MAPK family comprises three classic kinase pathways (ERK/p38/JNK).

The activation of the MAPK pathway is associated with lipid changes in some organisms. In the copepod *Paracyclopina nana*, BDE-47-induced oxidative stress, such as reactive oxygen species (ROS), mediates the activation of the Extracellular Signal-Regulated Kinase (ERK) and c-Jun-N-terminal Kinase (JNK) signaling cascades in the MAPK pathway. Subsequently, the activated MAPK pathway induces the binding of signal molecules to transcription factors (TFs) responsible for lipid synthesis, such as EcR, SREBP, and ChREBP promoters.

Additionally, stress stimuli lead to the conversion of saturated fatty acids (SFAs) to polyunsaturated fatty acids (PUFAs), a preparatory response observed in organisms adapting to stress. This process may be associated with the expression of elongase and desaturase genes (e.g., *ELO3*, Δ*5-DES*, Δ*9-DES*). These events further contribute to observed toxic phenotypic effects, including delayed early embryonic development and increased lipid droplet accumulation [68].

The MAPK pathway is intricately associated with cellular apoptosis in response to oxidative stress, with Reactive Oxygen Species (ROS) serving as a pivotal component for the activation of this signaling cascade. Exposure to BDE-47 induces alterations in the redox state of gonadal RTG-2 cells of *Oncorhynchus mykiss*, resulting in oxidative stress. A significant increase in malondialdehyde (MDA) levels and modifications in the intracellular GSH/GSSG ratio were observed. Enzymes associated with glutathione (GSH) underwent notable changes, and a substantial elevation in the mRNA levels of Nuclear Factor E2-Related Factor 2 (Nrf2) and its downstream genes related to antioxidant responses were noted. Simultaneously, activation of the p38 mitogen-activated protein kinase (MAPK) signaling pathway by BDE-47 was observed, further contributing to the induction of cellular apoptosis [45,75].

The MAPK pathway is also closely associated with the biological immune system. The activation of ERK is crucial for effective phagocytosis, blood cell movement, and lysosome protection. ERK and p38 phosphorylation play a pivotal role in bacterial killing. Wang et al. revealed the mechanism and pathway of stress response to BDE-47 in *Brachionus plicatilis* by transcriptomics, and found that the differentially expressed genes in ribosomes, estrogen signaling pathway responsible for repairing damage and inhibiting apoptosis were significantly down-regulated, whereas apoptosis-related genes related to the MAPK signaling pathway and apoptosis pathway were significantly upregulated, indicating that BDE-47 stress is able to induce rotifers to undergo apoptosis in rotifers [96]. Zhou et al. assessed the immune capability of blood cells exposed to BDE-47 in *Ruditapes philippinarum* and suggested that the decreased phosphorylation of ERK and p38 in the MAPK pathway, induced by BDE-47 in oysters, directly reduces the phagocytic activity and bacterial degradation capacity of blood cells, while increasing low-molecular-weight protein (LMP). Alternatively, the reduction in p-ERK may promote lysosomal membrane damage, indirectly impairing the immune capability of blood cells [70].

## 4. Conclusions and Future Prospects

Although the majority of polybrominated diphenyl ethers (PBDEs) have been prohibited from being discharged into water bodies due to the Stockholm Convention (United Nations Environment Programme, 2017) [2], their persistent nature results in their substantial retention within the marine environment. Furthermore, BDE-47, characterized as a persistent organic pollutant, accumulates significantly within marine organisms, leading to bioaccumulation and biomagnification along the food web. Tolerance levels and toxicological responses to the same substance often vary between marine and freshwater aquatic organisms. Advanced biological research techniques such as transcriptomics and other omics technologies are predominantly applied to study the toxicity mechanisms of BDE-47 in freshwater organisms, with rare applications in marine environments. This review comprehensively summarizes the toxic effects of BDE-47 on marine organisms, exploring potential molecular mechanisms, including oxidative stress, apoptosis induction through various pathways, activation of detoxification systems, metabolic disturbances, and the initiation of the MAPK pathway.

The toxic effects of BDE-47 on marine organisms’ merit further exploration, particularly in terms of sex-specific toxicity. Research suggests that BDE-47 appears to preferentially accumulate in the female bodies of marine fish (*Oryzias melastigma*) [79]. Currently, sex differences in detoxification systems, toxicity, metabolic mechanisms, and apoptotic pathways have been individually studied in a few marine species, but these toxic effects have not been integrated.

Additionally, studies have indicated that within organisms, BDE-47 undergoes not only debromination but also subsequent transformations into MeO-PBDEs and/or OH-PBDEs [97], which furthers’ rise to bioaccumulation [98], leading to varied accumulation levels across different organisms [99]. The sex-specific accumulation of hydroxylated or methoxylated BDE-47, coupled with its intergenerational transmission, has also been observed [100]. Therefore, exploring and summarizing differences between MeO-PBDEs and OH-PBDEs in other toxic effects, compared to the original BDE-47, are areas deserving further investigation.

Furthermore, BDE-47 undergoes alterations in morphology and toxicity when exposed to UV radiation in natural marine environments [101]. In seawater, it encounters other pollutants, and its toxic effects may be amplified or attenuated, especially in the presence of adsorbent micro-nano materials. This interaction may contribute to increased complexity in both the toxic effects and mechanisms [25,102]. It is important to note that BDE-47 is a highly lipophilic contaminant that tends to accumulate in lipid-rich biological tissues. When co-exposed with other novel pollutants, such as amphiphilic PFOA [93], its distribution pattern within the organism may change, potentially exacerbating its toxicity. Further exploration and comprehensive summarization of these aspects are warranted.

## Figures and Tables

**Table 1:** Lethal concentration of BDE-47 (LC_50_, expressed as average) evaluated in different marine organisms at different life stages.

Phylum	Species	Life Stage	Duration of Exposure	LC_50_ (µg/L)	Ref.
*Rotifera*	*Brachionus plicatilis*	0–2 h	48 h	2113 411	[38] [36]
			72 h	376	[38]
			96 h	163	[38]
*Arthropoda*	*Eurytemora pacifica*	0–2 h	96 h	57	[37]
	*Tigriopus japonicas*	0–2 h	96 h	851	[37]
	*Fenneropenaeus chinensis*	Post-larval juvenile shrimp	96 h	44.0	[36]
	*Acartia bifilosa*	BL = 0.08 ± 0.01 cm	48 h	341	[36]
*Mollusca*	*Argopecten irradians*	BL = 0.012 ± 0.002 cm	96 h	69.7	[36]
	*Sinonovacula constricta*	BW = 0.0051 ± 0.0005 g BL = 0.2 ± 0.05 cm	96 h	147	[36]
	*Crassostrea gigas*	egg	96 h	244.5	[39]
*Chordata*	*Takifugu rubripes*	BW = 0.042 ± 0.005 g BL = 1.0 ± 0.2 cm	96 h	387	[36]
	*Psetta maxima*	BW = 0.075 ± 0.007 g BL = 1.5 ± 0.2 cm	96 h	44.4	[36]

BL: the average body length; BW: the average body weight. h: hours.

## Data Availability

Data availability is not applicable to this article as no new data were created or analyzed in this study.
